# The Effect of Price Reduction on Salad Bar Purchases at a Corporate Cafeteria

**DOI:** 10.5888/pcd10.120214

**Published:** 2013-02-21

**Authors:** Thomas E. Kottke, Nicolaas P. Pronk, Abigail S. Katz, Juliana O. Tillema, Thomas J. Flottemesch

**Affiliations:** Author Affiliations: Nicolaas P. Pronk, Abigail S. Katz, Juliana O. Tillema, Thomas J. Flottemesch, HealthPartners Institute for Education and Research, Minneapolis, Minnesota.

## Abstract

The objective of this study was to determine the effect of a price reduction on salad bar purchases in a corporate cafeteria. We reduced the price of salad bar purchases by 50% during March 2012 and analyzed sales data by month for February through June 2012. We also conducted an anonymous survey. Salad bar sales by weight more than tripled during the price reduction and returned to baseline afterward. Survey respondents reported that the high price of salad relative to other choices is a barrier to purchases. Policies that make the price of salads equal to other choices in cafeterias may significantly increase healthful food consumption.

## Objective

HealthPartners is an integrated health system located in the upper Midwest and headquartered in Minneapolis, Minnesota (www.healthpartners.com/public); its mission and vision statements include a focus on healthy lifestyles. Although diets that include adequate amounts of fruits and vegetables are associated with good physical and good mental health (1,2), few respondents to a HealthPartners health assessment questionnaire reported eating adequate amounts (3). The objectives of this study were to test whether, and the extent to which, the decision to eat a salad for lunch by employees who eat at the HealthPartners corporate headquarters cafeteria was influenced by salad price.

## Methods

A commercial vendor provides food service at HealthPartners corporate headquarters. In March 2012, HealthPartners subsidized half of the price of lunchtime salad bar items (a reduction from $0.38 to $0.19 per ounce) so that salads cost approximately the same as entrees. The subsidy was publicized through an e-mail to the 2,643 employees on the headquarters building e-mail distribution list and by a large poster in the cafeteria.

Food sales were recorded by food category at the time of sale; aggregated data were available by month but not by day or week or by number of people making purchases. We compared mean daily sales in total and by food category for March with sales for February, April, May, and June. We also calculated salad bar sales by weight. We used the *t* test to determine whether salad bar sales in March differed significantly from sales in the other 4 study months. Price elasticity, defined as the percentage change in the quantity demanded for a product given a 1% change in product price(4), is a measure used in economics to show the responsiveness of consumers to changes in price. We calculated the price elasticity for salads.

In May 2012, we invited all employees on the headquarters e-mail distribution list to complete an anonymous online survey on their lunchtime eating patterns. The HealthPartners institutional review board did not require approval of the protocol because no data contained personal identifiers. 

## Results

Total daily sales by month for February, April, May, and June averaged $30,732 (standard deviation [SD], $1,523) and ranged from $29,095 in April to $32,699 in May. Daily salad bar sales for the months of February, April, May, and June averaged $3,687 (SD, $259). Daily salad bar sales in March averaged $6,747, or 83% higher than sales averaged for other months (*t*_4_ = 4.89; *P* = .008) (Table 1). Sales of less healthful options were somewhat but not markedly lower in March than in other months. The increase in salad bar sales by weight in March compared with other months was 366%, representing a price elasticity of 7.32.

**Table 1:** Effect of a Salad Bar Price Reduction in a Corporate Cafeteria, Minneapolis, Minnesota, 2012

Item	Total Daily Sales, $					
	February	March	April	May	June	5-Month Average (SD)
Breakfast grill	2,539	2,372	2,065	2,458	2,396	2,366 (180)
Bakery	2,599	2,697	2,638	2,848	2,778	2,712 (102)
Fresh fruit	854	838	656	873	788	802 (87)
Yogurt bar	451	470	494	558	537	502 (45)
Salad bar	3,344	6,747	3,629	3,899	3,874	4,299 (1,387)
Deli bar	2,281	1,941	2,364	2,424	2,411	2,284 (200)
Grab-n-go	930	800	924	2,303	1,085	1,208 (620)
Entree	4,105	3,438	4,022	4,092	4,083	3,948 (287)
Grill	1,739	1,465	1,584	1,548	1,588	1,585 (99)
Pizza	844	737	837	869	858	829 (53)
Exhibition	1,299	1,230	1,292	1,484	1,454	1,352 (111)
Desserts	608	737	658	761	716	696 (62)
Other products	1,180	857	942	1,114	1,029	1,024 (129)
Beverages	7,373	7,141	6,990	7,468	7,390	7,272 (200)
**Total**	**30,146**	**31,470**	**29,095**	**32,699**	**30,987**	**30,879 (1,360)**

Of 2,643 invitees, 677 (25.6%) responded to the survey. Only about one-third (236/677) of respondents reported they typically purchase salads at the cafeteria, but nearly all respondents reported they increased the number of times they purchased salads during March (Table 2). Responses to the open-ended question were nearly all positive; respondents asked that the price reduction continue and frequently cited the high cost of the salad bar relative to other lunch options as a barrier to eating salad for lunch.

**Table 2:** Employee Responses to an E-Mail Survey on Salad Bar Price Reduction in a Corporate Cafeteria, Minneapolis, Minnesota, 2012

Question (number of responses)	n (%)
**How many days a week are you usually at the office during lunchtime? (676)**	
1 d	19 (3)
2 d	34 (5)
3 d	68 (10)
4 d	176 (26)
5 d	379 (56)
**Do you normally eat salads for lunch when you are in the office? (677)**	
No	380 (56)
Yes	297 (44)
**How often during a given week do you eat salads for lunch when at the office? (295)**	
1 d	32 (11)
2 d	73 (25)
3 d	100 (34)
4 d	59 (20)
5 d	31 (11)
I don't eat salads for lunch	0
**Do you typically purchase salads at the cafeteria? (297)**	
No	61 (21)
Yes	236 (79)
**How many times during a given week do you typically purchase salads at the cafeteria? (236)**	
1 d	40 (17)
2 d	66 (28)
3 d	78 (33)
4 d	35 (15)
5 d	17 (7)
I don't eat salads for lunch	0
**During the salad bar special, did you increase the number of times you purchased a salad for lunch? (677)**	
No	97 (14)
Yes	580 (86)
**During the salad bar special, how often did you purchase a salad for lunch during a given week? (576)**	
1 d	63 (11)
2 d	157 (27)
3 d	169 (29)
4 d	121 (21)
5 d	66 (11)
**Would you continue to eat more salad if the price were permanently less? (578)**	
No	2 (0)
I'm unsure	21 (4)
Yes	555 (96)
**What are your thoughts about having a company subsidized salad bar at the cafeteria? (674)**	
I'm not in favor of it. I would not like to see a subsidized salad bar in the future.	15 (2)
I'm unsure or neutral.	67 (10)
I'm in favor of it. I would like to see a subsidized salad bar as a regular feature of Be Well.	592 (88)
**Since the salad bar special ended in March, have you decided to eat salad for lunch (673)**	
Less often	335 (50)
About the same	258 (38)
More often	80 (12)
**What else would you like to tell us about the salad bar?^a^ (359)**	
Cost is a barrier to eating salads	176
Offerings at the salad bar	71
Expression of appreciation for the reduced price	56
Arrangement of the salad bar	8
Other miscellaneous	48

a An open-ended question; comments were categorized.

## Discussion

Other trials showed similar results to ours. In 1 cafeteria intervention, reducing the price of salad by half increased salad consumption by 300% (5). An intervention that reduced the price of healthful choices in vending machines led to an 80% to 93% increase in purchases of low-fat snacks (6,7). A restaurant intervention showed a significant, albeit smaller, effect (8). These results contrast with a 2009 meta-analysis of 160 studies that reported that purchasing of fruits and vegetables for consumption at home is relatively inelastic — about 0.70 for fruit and 0.58 for vegetables (9). A US Department of Agriculture analysis also concluded that subsidizing fruits and vegetables in grocery stores would have little or no effect on consumption by low-income people (4).

These data suggest that price elasticity is low when consumers decide how much of a product to buy in a grocery store and can be high when they are dining in a restaurant or a cafeteria. It can also be high when they are selecting items from a vending machine. In grocery stores they appear to be asking, “How much product can I buy at the current price?” (a reflection of low elasticity), and in cafeterias they appear to be asking, “Which meal can I afford?” (a reflection of high elasticity).

Our data also suggest that consumers make a trade-off between eating healthful foods and using their cash in other ways. In the HealthPartners cafeteria, a typical lunch option is $4 to $5, and the price of a salad plate is about $8. Employees could select a salad at a premium of about $20 per week, or $600 to $1,000 per year, or they could select a less healthful option and have more money to take their family out to dinner or a movie. After a year, $600 to $1,000 could buy at least part of a family vacation. Although our survey data are limited by a 26% response rate, the survey data and the 366% increase in salad sales by weight in March suggest that cost is a significant barrier to salad consumption.

The change in purchases in response to the change of price suggests that salads purchased in a cafeteria are viewed as a luxury rather than a necessity (10). Our data, and the data of others, suggest that efforts to increase salad consumption by reducing price can be effective in cafeterias. Changing the price of salads could be done without affecting total revenues if the price of less healthful items were increased. In our study setting, because salad purchases are only approximately 15% of total sales, reducing the price by half could be fully subsidized by raising the price of other products by only approximately 9%. Such action would be easy to undertake by any corporation that hosts an employee cafeteria.
